# Prediction of constitutive A-to-I editing sites from human transcriptomes in the absence of genomic sequences

**DOI:** 10.1186/1471-2164-14-206

**Published:** 2013-03-27

**Authors:** Shanshan Zhu, Jian-Feng Xiang, Tian Chen, Ling-Ling Chen, Li Yang

**Affiliations:** 1 Key Laboratory of Computational Biology, CAS-MPG Partner Institute for Computational Biology, Shanghai Institutes for Biological Sciences, Chinese Academy of Sciences, Shanghai 200031, China; 2 State Key Laboratory of Molecular Biology, Institute of Biochemistry and Cell Biology, Shanghai Institutes for Biological Sciences, Chinese Academy of Sciences, Shanghai 200031, China

**Keywords:** RNA-seq, RNA editing, Potential SNP score, Constitutive editing, Editing box

## Abstract

**Background:**

Adenosine-to-inosine (A-to-I) RNA editing is recognized as a cellular mechanism for generating both RNA and protein diversity. Inosine base pairs with cytidine during reverse transcription and therefore appears as guanosine during sequencing of cDNA. Current approaches of RNA editing identification largely depend on the comparison between transcriptomes and genomic DNA (gDNA) sequencing datasets from the same individuals, and it has been challenging to identify editing candidates from transcriptomes in the absence of gDNA information.

**Results:**

We have developed a new strategy to accurately predict constitutive RNA editing sites from publicly available human RNA-seq datasets in the absence of relevant genomic sequences. Our approach establishes new parameters to increase the ability to map mismatches and to minimize sequencing/mapping errors and unreported genome variations. We identified 695 novel constitutive A-to-I editing sites that appear in clusters (named “editing boxes”) in multiple samples and which exhibit spatial and dynamic regulation across human tissues. Some of these editing boxes are enriched in non-repetitive regions lacking inverted repeat structures and contain an extremely high conversion frequency of As to Is. We validated a number of editing boxes in multiple human cell lines and confirmed that ADAR1 is responsible for the observed promiscuous editing events in non-repetitive regions, further expanding our knowledge of the catalytic substrate of A-to-I RNA editing by ADAR enzymes.

**Conclusions:**

The approach we present here provides a novel way of identifying A-to-I RNA editing events by analyzing only RNA-seq datasets. This method has allowed us to gain new insights into RNA editing and should also aid in the identification of more constitutive A-to-I editing sites from additional transcriptomes.

## Background

RNA editing is a post-transcriptional modification process which not only expands the number of functions encoded by our genomes but also provides additional mechanisms of gene regulation. The most predominant form of such editing in higher eukaryotes is adenosine-to-inosine (A-to-I) RNA editing, which is catalyzed by members of ADAR enzyme family (adenosine deaminases that act on RNA) [1,2]. The resulting inosines preferentially base pair with cytidines (C) and are therefore functionally guanosines (G), although there has been evidence that inosine can also pair with guanosine [3]. Thus, A-to-I editing can have profound effects on downstream RNA processing and function, including recoding of open reading frames, altering the pattern of alternative splicing, interfering with microRNA function, modulating RNAi activity, and playing other roles in gene regulation [1,2].

The pattern of A-to-I RNA editing, either site-specific or promiscuous, is likely to determine the fate of an edited RNA molecule. The majority of A-to-I editing in the human transcriptome is located within inverted-repeated Alu elements (IRAlus) positioned within introns and UTRs as revealed by the systematic comparison of cDNA or EST libraries to genomic sequences [4-7], and by genome-wide profiling of transcriptomes and genomic DNAs from the same individuals [8-10]. RNAs with extensively edited IRAlus within their 3′UTRs are retained in nuclear paraspeckles [11-13], although this retention is not always complete [12,14]. Compared to promiscuous A-to-I RNA editing in repetitive elements, site-specific editing in coding regions provides a rich source of genetic recoding that can influence protein function. The best-characterized editing sites in mammals occur in codons of pre-mRNAs encoding glutamate receptor B (GluR-B) and serotonin receptor 2C (5-HT_2C_R) [15,16]. In addition, site-specific A-to-I RNA editing outside coding sequences has been shown to interfere with miRNA pathways by affecting microprocessor or Dicer cleavage, RISC loading and mature miRNA function [17-22]. Thus, it is becoming increasingly apparent that A-to-I RNA editing plays important roles in regulating gene expression and product function.

Inosine base pairs with cytidine during reverse transcription and therefore appears as G during sequencing of cDNA. Thus, A-to-I editing sites can be inferred by the presence of G at a given position in a cDNA sequence but only A in the corresponding genomic position [1,2]. Most recently, the application of next-generation sequencing to cDNAs (RNA-seq) and genomic DNAs from the same human individual followed by extensive computational analyses revealed an additional large number of editing sites in both Alu and non-Alu elements [8-10]. Thus, the emergence of new technologies and approaches has enabled the identification of a growing list of editing sites.

Transcriptome and genomic DNA sequencing datasets are not always available for single individuals. However, RNA-seq data is widespread and available through public datasets and thus represents a relevantly rich source of yet unexplored RNA editing sites. There are two features that currently limit the application of RNA-seq data to identify A-to-I RNA editing without the relevant genomic information. On one hand, the nature of nucleotide mismatches reduces the ability to uniquely align RNA-seq reads to the genome, and therefore reduces the capability to retrieve nucleotide variants. On the other hand, true editing events are often hidden in a background noise caused by sequence errors, mapping errors and genome variations, including genomic single nucleotide polymorphisms (SNPs) and somatic mutations. Thus, it has been challenging to accurately identify editing candidates from transcriptomes in the absence of gDNA information.

To overcome the aforementioned issues, we have developed a new pipeline to accurately predict editing sites from 18 human RNA-seq datasets, even without knowledge of relevant genomic sequences from which the RNA-seq data were derived. We identified 2,245 constitutive A-to-I editing sites that occur in clusters (named “editing boxes”). Some of these are enriched in non-repetitive elements and exhibit an extremely high A-to-I conversion frequency. Importantly, editing sites located in non-repetitive editing boxes were validated in multiple human cell lines using conventional PCR and Sanger sequencing and were proven to be catalyzed by ADAR1. Finally, distinct editing ratios of RNA sites in editing boxes from different tissues/cell lines clearly suggest a spatial and dynamic regulation of A-to-I RNA editing across human tissues.

## Results

### A computational flow to predict clustered A-to-I editing sites from transcriptomes only

**Figure 1:**
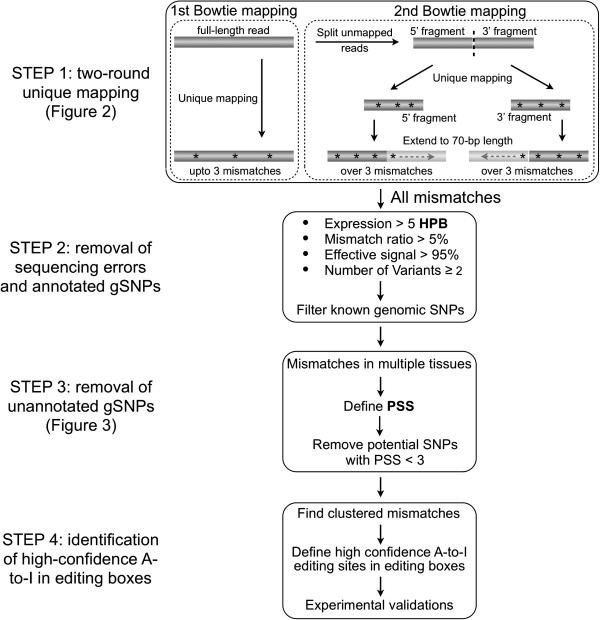
**A computational approach for the prediction of constitutive A-to-I editing sites in clusters from multiple RNA-seq datasets.** STEP **1**: Two-round unique mapping. STEP **2**: Removal of sequencing errors and annotated gSNPs. STEP **3**: Removal of unannotated gSNPs with customized PSS. STEP **4**: Identification of constitutive A-to-I editing sites clustered in editing boxes. See Materials and Methods for details.

It has been challenging to discover A-to-I RNA editing sites from RNA-seq datasets for a number of reasons. First, edited As are interpreted as Gs in sequencing reads. This leads to problems with alignment of edited reads to the genome. Second, random sequencing errors and mapping errors are often problematic. Third, some genomic polymorphisms and somatic mutations are unpredictable from an individual genome without knowledge of the genomic sequence. Finally, transcriptome and genomic DNA sequencing datasets are not always available for single individuals. To overcome these difficulties, we have developed a computational approach consisting of four key steps (Figure 1) to identify RNA editing from multiple RNA-seq datasets in the absence of the relevant genomic sequence.

**Figure 2:**
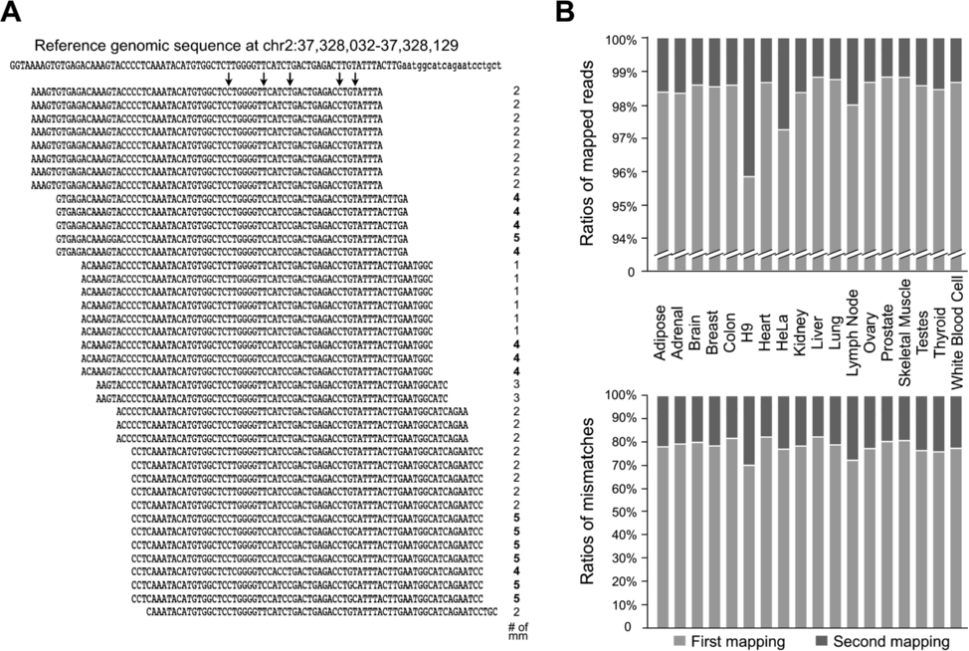
**A dramatic increase of nucleotide mismatch calling from a two-round unique mapping approach. **(**A**) Multiple mapped reads from RNA-seq data of human colon tissue were uniquely aligned to chr2: 37,328,032 -37,328,129 of the hg19 genome with the number of mismatches shown on the right. The predicted editing sites are highlighted with arrows. Reads with 4 and 5 mismatches (bold on right) were identified with the split and 2nd-round mapping approach and would have been missed with the default mapping. (**B**) The two-round mapping approach achieved a significant increase of mapped mismatches (bottom panel) and subtle changes of mapped reads (top panel). The 1st-round mapping, light grey bars; the split and 2nd-round mapping, dark grey bars.

STEP 1: a two-round unique mapping strategy with Bowtie to improve the mapping ability and to obtain an increased number of aligned mismatches. Multiple mapping pipelines have been developed to align individual RNA-seq reads to the corresponding genomes [23-26]. However, most mappers with default setting are not suitable to deal effectively with mismatches that result from RNA editing. To increase the mapping sensitivity to capture more mismatches, we applied a two-round-unique mapping with Bowtie to analyze 18 human cell line and tissue transcriptomes (Methods). As we found that both ends of sequence reads contain higher sequencing errors (Additional file 1), we trimmed 75-nt reads from both ends to 70-nt long for the first alignment. This mapping scheme allowed us to not only keep longer reads to map repetitive elements in the genome, but also retrieved a large number of mismatches. For instance, the second split-alignment resulted in only 1-4% of increased mapped reads compared with first alignments (Figure 2B, top panel, Additional file 2); however, the mapped mismatches were increased 20%-30% in different samples (Figure 2B, bottom panel). In addition, the application of this two-round mapping strategy with other aligners also dramatically increased the mismatch calling, but with a little increase in mapped reads (Additional file 3). Clearly, this two-round-unique mapping scheme significantly improved the alignment capability for mismatches, which in turn allowed us to obtain an accurate dataset of the editing site/ratio prediction and to identify previously unreported A-to-I conversions in human transcriptomes.

STEP 2: a series of stringent cutoffs to reduce sequencing/mapping errors and to remove known genomic SNPs. As different samples vary in genome coverage and sequencing depth, we used the HPB value (Additional file 4) to normalize the expression level for each transcribed site across samples, and selected a relatively higher cutoff at HPB > 5 for a given site, comparable to RPKM/FPKM > 5 for a gene, to call potential editing candidates in highly expressed sites. In our calculation, 5 HPB represented 8 ~ 19 raw hits for each base in different transcriptomes (Additional file 5). The relatively high HPB in our analysis allowed us not only to locate the position of an editing site, but also to accurately calculate the editing ratio of each site.

**Figure 3:**
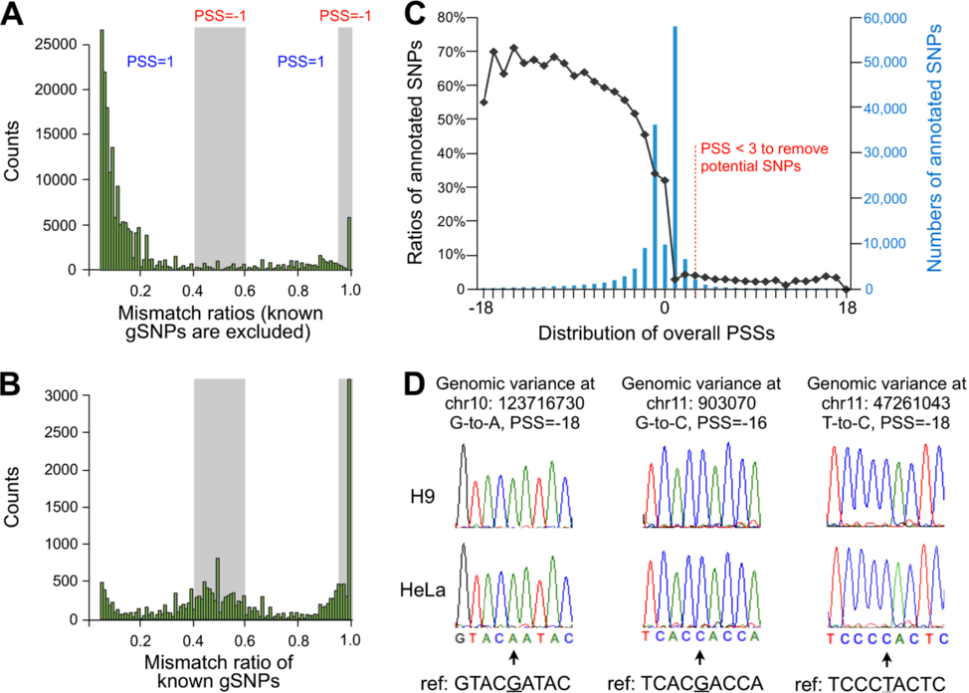
**Development and application of Potential SNP Scores (PSS) to filter out previously unannotated genome variations.** (**A**) The distribution of mismatch ratios of all non-gSNPs mismatches and (**B**) known gSNPs in H9 cells. PSS was given to test the possibility of a mismatch for either genomic variation (PSS = −1, with mismatch ratio ≥ 95% or between 40% ~ 60% in grey shadow) or editing (PSS = +1, with mismatches ratios between 5% ~ 40% or between 60% ~ 95%) in H9 cells. (**C**) Application of PSS to filter out previously unannotated genome variations. Over 97% of known genomic SNPs were filtered out with PSS cutoff at 3 (red dashed line). (**D**) Validation of previously unannotated genome variations predicted with PSS cutoff. Three examples with Sanger sequencing results were shown with their genomic locations, types of conversion and PSSs (full validation list is available in Additional file 6).

STEP 3: a new parameter, PSS, to remove unreported genomic variances by taking advantage of large numbers of RNA-seq datasets. PSSs for known SNPs were calculated using a similar strategy and their distribution was then plotted as a control (Figure 3C). From our analysis, 30% to 70% of mismatches carrying an overall PSS from −18 to 0 are known SNPs (black line with dots in Figure 3C), suggesting that the remaining mismatches carrying an overall PSS from −18 to 0 could be unreported genomic variations. Importantly, 100% (11 of 11) randomly picked mismatches with a PSS from −18, -16, or −11 were proven to be true genomic variations, but not editing events, by Sanger sequencing (Figure 3D and Additional file 6). On the other hand, only less than 5% of mismatches carrying an overall PSS from 1 to 18 are known SNPs, suggesting that we could remove over 95% of reported and unreported genomic variations with a PSS ≥1 (Figure 3C). However, given the fact that there are a large amount of known gSNPs carrying PSS at −2 to 2 (blue histogram in Figure 3C), in the current analysis, we set up a even more stringent cutoff to remove potential genomic variation sites with PSS < 3, which filtered out over 97% expressed SNPs (red line in Figure 3C). From the data we noted that some well-characterized editing sites were found in a tissue-specific manner. For example, Q/R and R/G sites in the pre-mRNA of GluR-B were detected only in brain with the expected editing frequencies (Additional file 7A). These tissue-specific editing events were largely due to the brain-specific expression of GluR-B RNAs (Additional file 7B). In the current study, we focus on editing sites constitutively detected from multiple human tissues (constitutive editing sites), and tissue-specific expressed RNAs and editing events (tissue/cell-specific editing sites) were not considered.

STEP 4: predicting constitutive A-to-I editing sites that occur in clusters. Owing to the absence of relative genomic sequence information with which to compare RNA sequence data, we enriched high confidence A-to-I editing sites by considering the fact that A-to-I sites could be clustered or promiscuously edited within specific genomic regions. In our analysis, we found that many A-to-G/T-to-C sites, but few from other types of nucleotide conversions or known SNPs, could be clustered (Table 1B and 1C). In addition, we further performed strand specific RNA-seq with RNAs collected from H9 cells and found that 100% of the identified T-to-C sites were transcribed from “-” strand of chromosome and A-to-G sites were from “+” strand of chromosome in H9 cells, suggesting clustered A-to-G/T-to-C mismatches were most likely to be true A-to-I editing. As they were detected from no less than three transcriptomes, we classified these A-to-G/T-to-C mismatches after STEP 4 as constitutive A-to-I sites, and named regions containing such sites as “editing boxes”.

**Table 1:** Characterization of editing prediction pipeline

**A**				
		**# of all m.m. in *Alu***	**# of A-to-I in *Alu***	**A-to-I ration in all m.m.**
STEP2: w/o PSS cutoff		95,187	57,502	60.41%
STEP3: PSS cutoff		8,721	7,266	83.32%
STEP4: in editing box		1,995	1,995	100%
**B**				
		***Alu***	**Non-*Alu *repetitive**	**Non-repetitive**
**A-to-I**	Editing boxes (sites)	238 (1995)	7 (61)	21 (189)
	Ave. length (nt)	~108 nt	~86 nt	~71 nt
	Ave. conversion rate of As to Is	~31%	~40%	~51%
A-to-C		0	0	2 [14]
T-to-G		0	0	1 [6]
A-to-T		0	0	0
T-to-A		0	0	0
C-to-A		0	1 [6]	1 [5]^*^
G-to-T		0	0	1 [5]
C-to-G		0	0	0
G-to-C		0	0	1 [5]^*^
C-to-T		0	0	1 [5]
G-to-A		0	0	1 [6]^*^
**C**				
		**in IR *AIu *s**	**within 1 kb to IR *AIu *s**	**> 1 kb to IR *AIu *s**
**A-to-I editing boxes (sites)**		208 (1763)	36 (310)	24 (172)

(A) The enrichment of A-to-I conversion in Alu elements after each step of our computational flow. (B) Editing boxes/sites distribution in Alu, non-Alu repetitive and non-repetitive regions. Asterisk indicated non A-to-Gs (noncanonical editing) sites are validated to be false positives. (C) Editing boxes/sites distribution in IRAlus, within 1 kb to IRAlus and over 1 kb to IRAlus.

Since it is known that A-to-I editing sites are enriched in Alu elements, we calculated the enrichment of A-to-I conversion in Alu elements after each step of our computational flow. As shown in Table 1A, ~ 60% mismatches in Alu elements were A-to-G/T-to-C conversions after STEP 2, compared to ~ 24% before STEP 2 (data not shown). Furthermore, ~ 83% mismatches in Alu elements were A-to-G/T-to-C conversions after PSS cutoff, indicating PSS could greatly improve the identification of true editing sites. Finally, 100% mismatches identified in Alu elements were A-to-Is after the cluster filtering, while several regions clustered with other types of nucleotide conversions failed to be validated with Sanger sequencing (Table 1B and data not shown).

In total, we identified 2245 constitutive A-to-I editing sites clustered in 266 editing boxes (Additional file 5). Although the editing boxes were largely from Alu elements, we found 7 editing boxes from non-Alu repetitive regions and 21 editing boxes from non-repetitive regions (Table 1B). The average length of non-repetitive editing boxes is 71 nt, which is shorter than that of Alu and non-Alu repetitive editing boxes (Table 1B). However, the average A-to-I nucleotide conversion rate in non-repetitive editing boxes is about 51% of all As, which is higher than Alu and non-Alu repetitive editing boxes (Table 1B), suggesting the surprising result that promiscuous A-to-I editing can occur in non-repetitive regions.

### Characterization of predicted constitutive A-to-I sites in editing boxes

**Figure 4:**
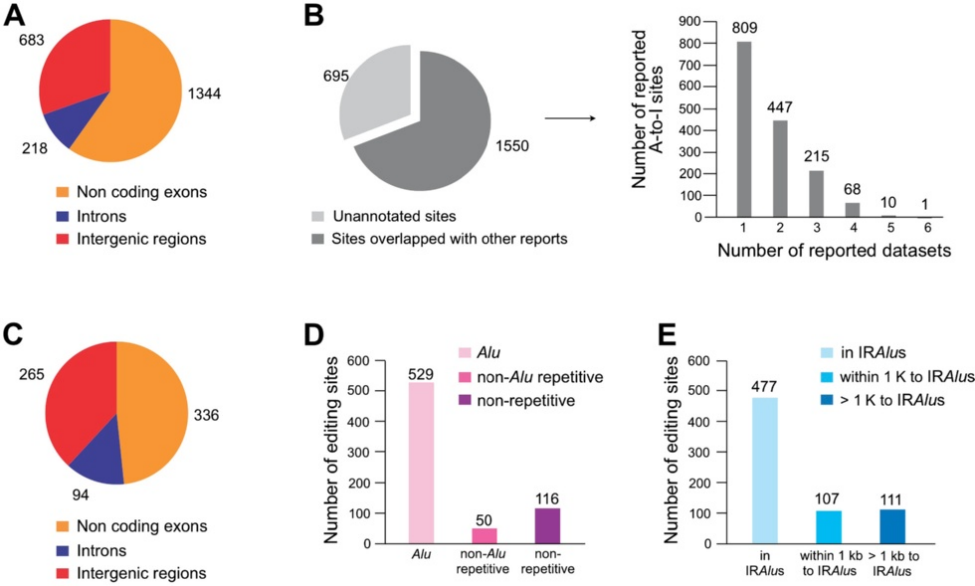
**Characterization of RNA editing sites in editing boxes.** (**A**) The genomic distribution of constitutive A-to-I editing box sites. (**B**) Comparison of predicted constitutive editing box sites with other studies [8-10,27] and DARNED database. 695 (about 31%) constitutive editing sites in clusters were previously unreported, compared with 1550 (69%) sites were overlapped with at least one dataset (left panel). Only a few sites were reported from multiple datasets (right panel). (**C**) The genomic distribution of newly identified editing box sites. (**E**) The distribution of newly identified editing box sites in IRAlus, within or over 1 kb to IRAlus regions. (**D**) The distribution of newly identified editing box sites in Alu, non-Alu repetitive or non-repetitive regions.

Unlike tissue-specific editing, all 2,245 A-to-I sites in editing boxes identified in this study were constitutive editing sites that existed in multiple tissues/cell lines. These editing sites are all located in noncoding regions, with the majority in noncoding exons and intergenic regions and ~10% in introns (Figure 4A). Compared with several other recent studies [8-10,27] and DARNED database (Figure 4), 1550 editing sites (69%) were reported in at least one dataset and 695 (31%) were novel sites (Figure 4B, left panel). More interestingly, 809 reported editing sites were found in only one of the six datasets, and only one site was present in all six datasets (Figure 4B, right panel). The huge difference among these datasets could be due to a variety of cells/tissues used in individual studies as well as different computational approaches in acquiring editing sites. These comparisons also suggested that our computational flow allowed us to efficiently predict A-to-I editing sites across transcriptomes even without the support of relevant genomic information.

We further examined genomic locations of 695 new editing sites in editing boxes. These new sites are located in noncoding regions, including noncoding exons, intergenic regions and introns (Figure 4C). In addition, many editing sites in intergenic regions were located within 10 kb of annotated genes, suggesting these unannotated regions could be extended 3′-UTRs of adjacent genes. Although editing box sites were largely from Alu elements, 50 and 116 editing box sites were from non-Alu repetitive or non-repetitive regions, respectively (Figure 4D). Additional analyses revealed that the majority of these editing boxes were located in or close to IRAlus (within 1 kb to IRAlus) (Table 1C), suggesting promiscuous editing in non-Alu editing boxes could be facilitated by the recruitment of ADAR enzymes to nearby duplex structures. However, 111 new editing sites in non-repetitive regions (from 172 in total, Table 1C) were further than 1 kb from the nearest IRAlus (Figure 4E), suggesting that other mechanisms may be involved in these promiscuous editing events.

### Predicted constitutive A-to-I sites from non-repetitive editing boxes are catalyzed by ADAR1

It is known that the majority of A-to-I editing in the human transcriptome occurs within Alu elements [4-6,8-10,27]; however, it was unexpected to identify promiscuous editing sites in non-repetitive sequences. Thus, we randomly selected several such editing boxes for validation.

**Figure 5:**
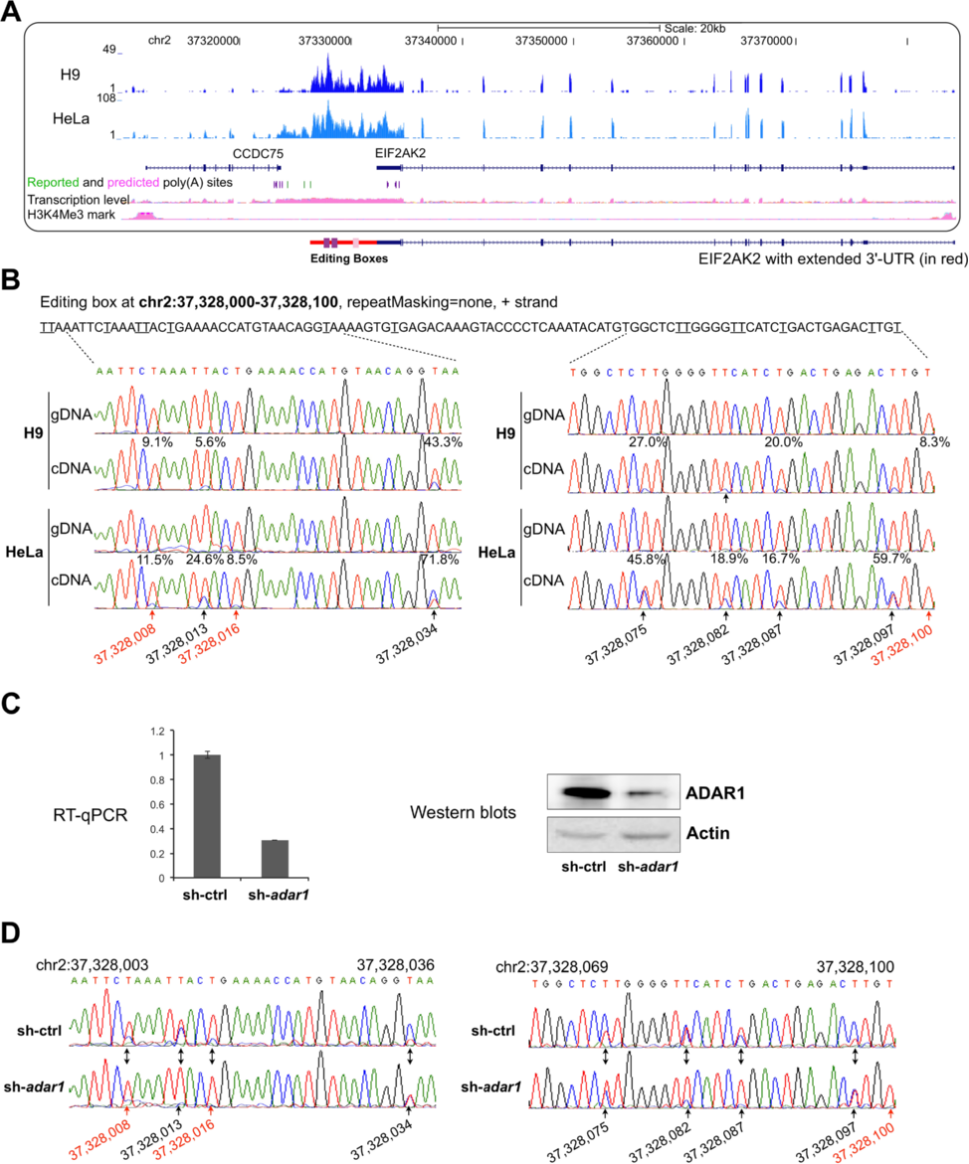
**Validation of constitutive A-to-I sites in non-repetitive editing boxes.** (**A**) Three editing boxes were identified within an intergenic region at chromosome 2. A screenshot from the UCSC genome browser for its sequencing signals in H9 cell (dark blue) and HeLa cell (light blue) with annotated gene models (exons in thick dark blue bars, introns labeled with arrowheads as transcription direction) was shown. CCDC75 is transcribed from the plus strand while EIF2AK2 is transcribed from the minus strand of chromosome. A new gene model of EIF2AK2 with extended 3^′^ UTR (red line) is drawn beneath the UCSC genome browser snapshot box. Two editing boxes in non-repetitive regions (purple bars) are located in the extended 3^′^ UTR region together with another editing box in Alu (pink bar). (**B**) Validation of constitutive A-to-I editing sites. Predicted A-to-I editing sites were indicated with underlines (shown as T-to-Cs on plus strand of chr2), and their predicted editing ratios were shown above each site in the cDNA sequencing chromatograms. Novel editing sites were highlighted with red arrows and their genomic sites were indicated in the bottom, reported sites were in black. (**C**) Knocking down of adar1 in HeLa cells with shRNA. Both RT-qPCR (left panel) and Western blots (right panel) showed a successful ADAR1 knockdown (sh-adar1) compared with a scramble shRNA (sh-ctrl). (**D**) Newly identified promiscuous A-to-I editing sites in non-Alu elements are catalyzed by ADAR1.

In an intergenic region between genes CCDC75 and EIF2AK2 in chromosome 2, two non-repetitive editing boxes (purple bars in Figure 5A) and one Alu editing box (one of IRAlus, pink bar in Figure 5A) are separated by over 1 kb. We found that this intergenic region is differentially expressed in all examined cell lines/tissues (Additional file 8). We further checked epigenetic modifications of ChIP-Seq analysis from ENCODE project, but these showed no signs of active transcription starts adjacent to this region, suggesting this intergenic region is more likely co-expressed with its neighboring gene(s). More careful analysis revealed that similar expression signals were detected in the intergenic region with EIF2AK2, and stopped at a reported (blue bars) poly(A) site in H9 cells, suggesting this intergenic region is an extended 3′ UTR of EIF2AK2. This was further confirmed by strand specific RNA-seq in H9 cells.

The validation results from gDNAs and cDNAs of both H9 and HeLa cells for the two editing boxes from non-repetitive regions revealed a high correlation with our bioinformatic predictions. Sites predicted to be edited in H9 and/or HeLa cells (Table 2) with over 10% editing ratios were validated by Sanger sequences (Figure 5B and Additional file 9A-9C). In addition, the estimated editing ratios by the two methods correlate relatively well (r = 0.845), as indicated by Additional file 9D. Taken together, these results suggested that our predicted editing sites in editing boxes are highly confident. Moreover, knockdown of ADAR1 (Figure 5C) significantly reduced editing ratio of individual A-to-I sites in editing boxes (Figure 5D and Additional file 10), suggesting that editing in non-repetitive editing boxes is catalyzed by ADAR1.

**Table 2:** Editing ratios of constitutive A-to-I sites at one editing box in 18 human samples

**chr2:**	**37,328,008**	**37,328,012**	**37,328,013**	**37,328,016**	**37,328,034**	**37,328,075**	**37,328,082**	**37,328,087**	**37,328,100**
H9	9.1%		5.6%		43.3%	26.9%		20.0%	8.3%
HeLa	11.5%		24.7%	8.5%	71.8%	45.8%	18.9%	16.7%	
Adipose	30.2%		41.5%	20.7%	78.0%	74.2%	63.3%	38.1%	8.8%
Adrenal	41.9%	7.5%	32.3%	25.9%	87.8%	69.5%	53.0%	38.6%	
Brain	24.3%	5.6%	42.3%	28.0%	87.2%	63.8%	46.8%	26.6%	14.6%
Breast	9.7%		14.2%	15.5%	63.8%	47.0%	50.0%	12.5%	
Colon	19.3%		19.6%	18.1%	91.8%	50.8%	7.0%	26.9%	
Heart	9.1%		18.4%		53.3%	13.3%	17.6%	18.4%	
Kidney	22.4%	6.4%	29.1%		58.9%	45.9%		15.1%	6.5%
Liver		50.0%	54.2%		76.0%				
Lung	26.4%	7.8%	19.6%	13.7%	59.5%				29.3%
Lymph Node			29.8%	14.3%	65.7%	39.1%	20.0%	48.6%	
Ovary	11.2%		40.1%	15.4%	71.2%	32.5%	25.8%	23.5%	
Prostate	27.8%	7.9%	43.4%	30.3%	79.7%	95.6%	46.7%	33.2%	11.4%
Skeletal Muscle			33.3%		20.9%				
Testes	19.6%		16.1%	15.5%	89.0%	42.6%	38.8%	31.1%	5.5%
Thyroid	13.2%	8.2%	31.8%	20.3%	64.8%	17.6%	28.8%	32.0%	
White Bllod Cell	7.6%		31.1%	24.5%	66.3%	26.3%	36.7%		
PSS	13	5	8	13	10	3	5	12	7
Darned database^a^			Yes		Yes	Yes	Yes		
Li, *et al*. 2009^24^			Yes		Yes	Yes	Yes		
Bahn, *et al*. 2012-BC^9^			Yes		Yes	Yes	Yes	Yes	
Bahn, *et al*. 2012-U87MG^9^					Yes	Yes			
Peng, *et al*. 2012^8^			Yes						
Ramaswami, *et al*. 2012^10^			Yes		Yes	Yes	Yes		

Predicted editing ratios of nine A-to-I sites in editing box at chr2: 37,328,008 -37,328,100 are listed in all examined cell lines/tissues. Blank indicates either no editing or failure of passing our stringent cutoffs (HPB > 5, etc.). Five annotated sites were reported from different datasets/analyses. ^a^Dataset of RNA editing at http://darned.ucc.ie/.

Since the filtering applied in this study achieved high accuracy (100% validation) in predicting clustered A-to-I editing sites, we also investigated the performance of this method on editing sites that are not clustered (Table 3). However, only about half of randomly selected predicted sites could be experimentally validated in both H9 and HeLa cells (7 out of 15, Table 3). This further indicated that our method is more reliable for prediction of clustered A-to-I editing sites than for non-clustered ones in the absence of the relevant genomic sequences.

**Table 3:** Comparison of predicted clustered and non-clustered constitutive A-to-I sites

**A**						
	**A-to-I conversions**		**A-to-Is in H9 and HeLa**		**Numbers of validated sites**	
Clustered sites	2,245		296		22 of 22	
Non-clustered sites	10,220		1,542		7 of 15	
**B**						
Predicted A-to-I sites			Predicted A-to-I ratios		Validation	
Chr. location	Altered base	Gene location	H9	HeLa	H9	HeLa
chr1:40041484	A- > G (+)	Coding	28.1%	29.5%	−	−
chr4:184186228	A- > G (+)	Coding	30.8%	28.1%	−	−
chr6:159187882	A- > G (+)	3^′^UTR	46.2%	50%	−	−
chr7:44841489	A- > G (+)	3^′^UTR	78.4%	69.2%	+	+
chr8:48890109	A- > G (+)	3^′^UTR	29.6%	32.5%	+	+
chr10:75008955	A- > G (−)	3^′^UTR	59.5%	65.8%	+	+
chr17:4068050	A- > G (+)	3^′^UTR	31.8%	31.7%	−	−
chr17:61898921	A- > G (−)	Coding	27.5%	16.9%	+	+
chr17:80445942	A- > G (+)	Coding	34.4%	32.1%	−	−
chr19:10755103	A- > G (−)	3^′^UTR	95.3%	96.7%	−	−
chr19:34718735	A- > G (+)	3^′^UTR	46.5%	27.9%	+	+
chr19:39874895	A- > G (+)	3^′^UTR	36.1%	38.9%	−	−
chr20:30253695	A- > G (+)	3^′^UTR	20.0%	29.9%	−	−
chrX:54589730	A- > G (+)	3^′^UTR	30.0%	46.3%	+	+
chrX:54589774	A- > G (+)	3^′^UTR	6.9%	8.8%	+	+

(A) Comparison of clustered and non-clustered constitutive A-to-I sites identified with or without STEP4 cutoff. Editing sites detected in HeLa and H9 cells were further used for validation with Sanger sequencing. (B) Validation results of randomly selected non-clustered A-to-I editing sites. “+”, validated to be an editing site; “-”, validated not to be an editing site.

### Characterization of promiscuous A-to-I RNA editing from non-repetitive editing boxes

**Figure 6:**
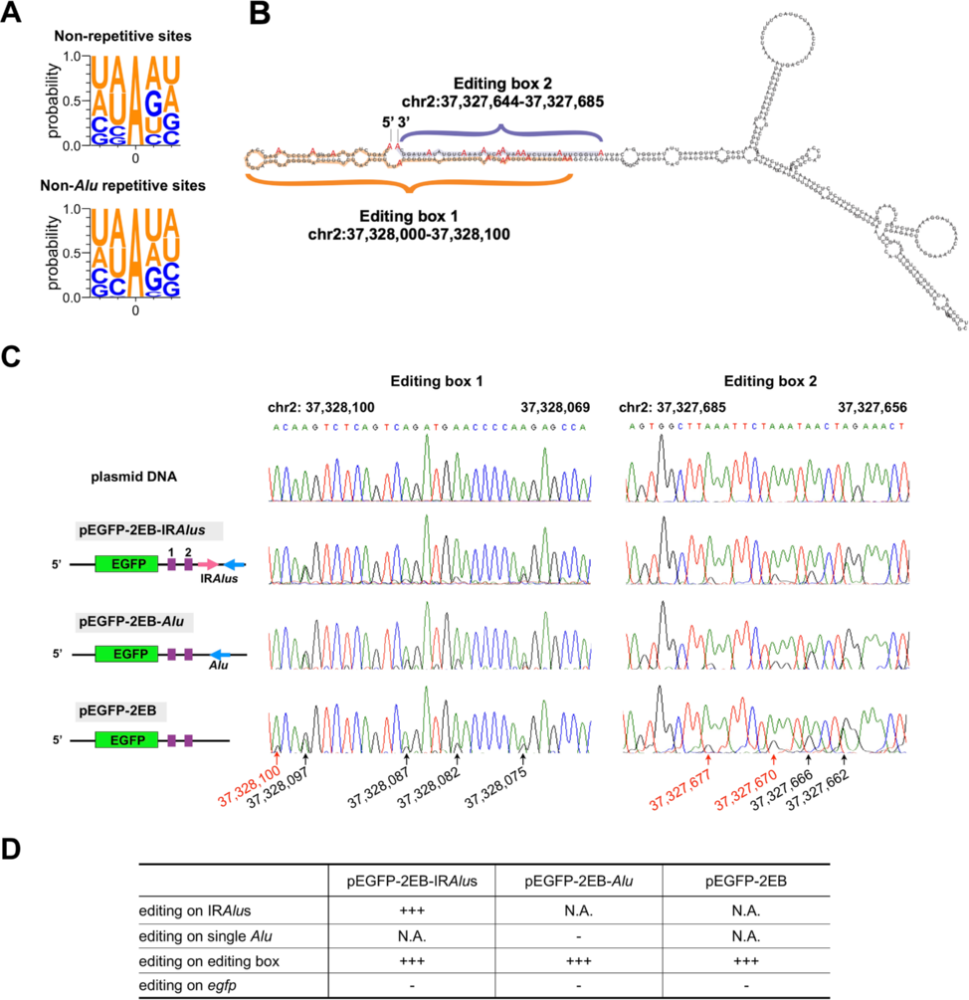
**Characterization of promiscuous A-to-I RNA editing clustered in non-repetitive editing boxes.** (**A**) Neighbor preferences of A-to-I RNA editing clustered in non-repetitive or non-Alu repetitive editing boxes. Site “0” indicates the editing sites. Probabilities of two upstream and two downstream nucleotides are indicated. (**B**) Structure prediction suggests a dsRNA duplex of two editing boxes in chr2. Genomic locations of two adjacent editing boxes are highlighted by different colors. (**C**) Editing of editing boxes is independent of adjacent IRAlus. Sequences with editing boxes were cloned into 3^′^UTR of egfp mRNA with a pair of adjacent IRAlus (pEGFP-2 EB-IRAlus), a single Alu (pEGFP-2 EB-Alu) or non-Alu (pEGFP-2 EB). (**D**) Editing levels in IRAlus, single Alu and editing boxes of each transfected plasmid shown in (**C**). “+++”, extensive editing; “-”, low editing; “N.A.”, not detected.

Since this work is the first demonstration of promiscuous editing in non-repetitive regions catalyzed by ADAR1 (Table 1 and Figure 5), we further characterized these sites in greater detail. Although there were no consensus sequences in all non-repetitive editing boxes, we found that ADAR1 preferentially targets adenosines when the 5′ nearest neighbor is A ≈ U > C > G (Figure 6A). This is in the agreement with known neighbor preferences of ADAR1 enzyme, but is slightly different from recently refined predicting sites of ADAR editing for an ~800 bp dsRNA (U > A > C > G) [28]. Moreover, structure prediction revealed that some of such editing boxes could potentially form long dsRNA duplexes with adjacent sequences (Figure 6B), suggesting the promiscuous A-to-I RNA editing in non-repetitive editing boxes may involve a mechanism similar to that of IRAlus. However, since over 90% of these editing boxes were located in or close to IRAlus, we could not exclude the possibility that their editing is coupled to the recruitment of ADAR enzymes to nearby Alu-related duplex structures [29].

To further test this possibility, we cloned sequences of editing boxes in 3′UTR of egfp or in the upstream region of single Alu or IRAlus in 3′UTR of egfp (Figure 6C). We have previously shown that IRAlus, but not single Alus, can be extensively edited when expressed from plasmid vectors, even during transient transfection [12]. We reasoned that if the adjacent IRAlus recruit ADARs to the nearby editing boxes, we would find more editing sites in editing boxes in vector containing IRAlus than those containing single Alu or no Alu. Otherwise, if editing boxes alone are sufficient to recruit ADARs, we would observe promiscuous editing in all examined vectors. Strikingly, our analyses revealed that sequences in editing boxes in all examined vectors were extensively edited in a similar way as that observed in their endogenous loci (Figure 6C and 6D). These results demonstrated that non-repetitive editing boxes alone can be edited by ADAR1, independent of adjacent IRAlus.

### Constitutive A-to-I sites in editing boxes are highly dynamic across human tissues

**Figure 7:**
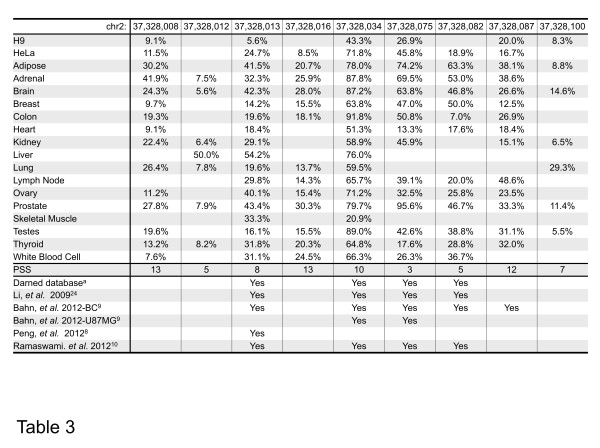
**Highly dynamic regulation of A-to-I editing in editing boxes across human tissues/cell lines.** Editing ratios of two non-repetitive (purple) and one Alu (pink) editing boxes (shown in Figure 5A) were marked with colored histograms for each site in H9 cell, HeLa cell, Adipose and Brain. The colored dots represent no report of editing events due to the stringent cutoffs. Full dataset for these editing boxes were available in Additional file 11.

As 2,245 constitutive A-to-I sites could be found in multiple human tissues and cell lines, we were able to analyze the spatial and dynamic regulation of A-to-I RNA editing. Surprisingly, constitutive A-to-I sites in editing boxes are highly dynamic across human tissues at two levels. On one hand, individual sites exhibit distinct patterns of editing across human tissues and cell lines (Table 2 and Figure 7). On the other hand, the editing efficiency of closely located editing boxes is highly dynamic. Interestingly, non-repetitive editing boxes (Figure 7, purple histograms, Table 2 and Additional file 11) exhibited even more striking differences than editing boxes of IRAlus (Figure 7, pink histograms) among examined samples. This indicated that different mechanisms could facilitate promiscuous editing within the same genomic characteristics in different tissues/cell lines and that ADAR editing is affected by more than nearest neighbors and local RNA structures (Figure 6).

Taken together, we have developed an approach to quantitatively profile constitutive A-to-I RNA editing from multiple human transcriptomes in the absence of the relevant genomic information. The application of our approach has allowed us to identify a large number of clustered constitutive A-to-I sites, including 695 novel sites. Our analysis also revealed that non-repetitive editing boxes could be promiscuously edited by ADAR1, independent of their adjacent IRAlus. Finally, although functionally unknown, marked differences of editing ratios in the same sites identified in editing boxes clearly suggest a spatial and dynamic regulation of A-to-I RNA editing across human tissues.

## Discussion

RNA-seq datasets, widespread through currently available public databases, are rich sources to search for A-to-I RNA editing sites. However, RNA-DNA mismatches between RNA-seq reads and the genome make the alignment of nucleotide variations to the genome problematic. In addition, transcriptome and genomic DNA sequencing datasets are not always available for single individuals, thus making straightforward prediction of A-to-I editing sites from available transcriptomes even more challenging. In this study, we developed a new computational approach to predict RNA editing from multiple tissues in the absence of the genome information. An additional 695 novel A-to-I editing sites have been identified compared to several other recent studies [8-10,27] and DARNED database (Figure 4B). We expect to detect more constitutive A-to-I RNA editing sites with additional sets of human transcriptomes as inputs by obtaining a higher PSS value for each A-to-G mismatch site. In addition, discrepancies of reported editing sites could be due to a variety of cell lines/tissues used in different studies (Figure 4B) [8-10,27].

Very recently, Ramaswami *et al*. also reported the identification of edited sites from transcriptome data only [30]. Their method was reported earlier [10] and slightly modified for identifying RNA editing sites in the absence of the related genomic DNA sequencing datasets [30]. In our present study, the pipeline was designed to identify clustered and constitutively edited A-to-Is. In total, 2,245 such editing sites were identified, including 695 new ones. Strikingly, these new sites were still largely missed by Ramaswami et al. [30] although much larger datasets were used. For example, they identified 181 out of 695 from 40 human lymphoblastoid cell lines, 273 out of 695 from 50 human brain samples, and 339 out of 695 from the same 16 human tissue samples.

Since we focused on clustered A-to-Is which are constitutive edited in at least three human tissues/transcriptomes, limited editing sites were identified in this study. It is also noteworthy that some limitations exist in this approach, including the insufficiency to predict more restricted tissue-specific editing, the inadequacy to identify some true editing sites with 40-60% or >95% editing ratios, and inaccuracies in identifying non-clustered editing sites (about 47% experimental validation, Table 3). For instance, true editing sites, such as A-to-I sites in pre-mRNAs of GluR-B, were not addressed in our study. In addition, true editing sites with low expression or low editing ratios could have been missed due to stringent cutoffs in the computational flow. These true editing sites would be captured if multiple RNA-seq datasets from the same tissue (to achieve a higher PSS value) and higher depths of RNA-seq datasets from individual samples were included in the future analysis. While a few non A-to-Gs (noncanonical editing) sites might be expected, none could be validated as true editing sites. These noncanonical sites could be derived mostly from mis-mapping reads to a highly similar genomic duplicate region, as suggested by Piskol et al. [31]. In the future, more stringent filters are needed for RNA editing prediction to remove this type of mapping errors.

Strikingly, we found that promiscuous RNA editing is not restricted to transcribed inversely orientated repetitive elements, such as IRAlus. Our analysis revealed many predicted constitutive A-to-I editing sites that appeared in clusters and were enriched in non-repetitive editing boxes with an extremely high A-to-I conversion frequency (Table 1B). A recent study suggested that editing of non-Alu sites appeared to be dependent on nearby edited Alu sites, likely by the recruitment of ADAR enzymes to nearby duplex structures [10]. However, we demonstrated that editing boxes alone were sufficient to be edited promiscuously by ADAR1 in expression vectors, and adjacent IRAlus have little effect to facilitate more editing (Figure 6). Although we could identify no consensus sequences in non-repetitive editing boxes, they are likely to form dsRNAs and the edited sites have similar 5′ neighbor preferences as reported recently for other ADAR1 substrates [28]. Thus, these new substrates predicted in this study further expanded our knowledge of the catalytic pattern of A-to-I RNA editing by ADAR1.

## Methods

### RNA-seq datasets

RNA-seq datasets from 16 human tissues sequenced by Illumina HiSeq 2000 (Illumina Human Body Map 2.0 Project) and two additional cell lines sequenced by Illumina Genome Analyzer IIx (GAIIx) [32] were retrieved from Gene Expression Omnibus (GEO:GSE30611 for tissues and GEO:GSE24399 for cell lines). About 40 ~ 80 million 75-nt single reads from each poly(A) + RNA-seq sample were obtained and further trimmed to 70-nt long at both 5′ and 3′ ends for 2 nt and 3 nt, respectively to reduce high sequencing errors at read ends (Additional file 1).

### Customized mapping strategy (STEP 1)

A two-round-unique mapping strategy with Bowtie [23], SOAP [8], or BWA [9] was applied to retrieve an increased number of mismatch calling (Figure 1). First Bowtie (v 0.12.8) mapping was performed from 70-bp reads to the hg19 human genome/junction [32] with up to three mismatches. After removal of multiple-aligned reads, unmapped 70-bp reads were split into two 35-nt fragments. 35-nt fragments from 5′ and 3′ were sequentially applied for the second unique mapping with up to three mismatches. The mapped 35-nt fragments were then extended to the other half with no more than 6 mismatches in total. In addition, reads with a distribution bias of mismatches that indicate higher sequencing errors at read ends are also excluded in this analysis. Other aligners (like BWA) can certainly be used for analysis directly with high mismatch allowance, but new parameters are needed to avoid/remove sequencing and mapping errors. The split scheme allowed us to retrieve more mismatches (up to six editing sites within 70-nt compared with three in default), and improved our capability in identifying the clustered RNA editing sites (Figure 2).

### Removal of sequencing errors and annotated gSNPs (STEP 2)

As the strand information of these RNA-seq datasets was not available, we referred plus strand of (“+”) chromosomes as reference for mismatch calling. In addition to trim 75-nt reads from both ends to 70-nt, we carried out the following stringent criteria for mismatch calling: (i): Each mismatch site must have a Hits Per Billion-mapped-bases (HPB) > 5. Since multiple RNA-seq datasets with different sequencing depths were used in this study, we developed HPB to normalize the expression level for each base across samples, and selected a HPB > 5 for each mismatch site (comparable to RPKM/FPKM > 5 for genes, Additional file 4) to focus on highly expressed mismatches. (ii): To improve the predicted editing accuracy and reduce false positives, we used mismatch ratio > 5% as a cutoff. Mismatch ratios were calculated by using mismatched hits vs all hits on the same sites. For example, G:(A + C + G + T + N) > 5% for A-to-G mismatch in a corresponding genomic position as A, and etc. (iii): To reduce random sequencing errors and to improve the correct assignment of sequence reads, we used effective signal > 95% as a cutoff. For example G:(C + G + T + N) > 95% for A-to-G mismatch, and etc. (iv): Require at least two individual reads with the same type of nucleotide conversion. (v): We finally filtered out gSNPs from the common SNP database (build 135, http://hgdownload.cse.ucsc.edu/goldenPath/hg19/database/snp135Common.txt.gz) and 1000 Genome database (http://evs.gs.washington.edu/EVS/, downloaded on July 15, 2012).

### Removal of unannotated gSNPs by customized PSS (STEP 3)

PSS was set up to further reduce unknown genomic noise by taking advantage of multiple human tissue RNA-seq datasets. Notably, most mismatches showed low ratios (< 20%) from multiple human tissues, while some showed high mismatch ratios (>60%) (Figure 3A, and Additional file 12). In contrast, mismatch ratios of known gSNPs were significantly enriched in two peaks: one major peak at around 100% (homozygous) and a minor peak at around 50% (heterozygous) mismatch ratio (Figure 3B, Additional file 12). Theoretically, genomic variations would give rise to either ~ 50% or ~ 100% mismatch ratios depending on whether the variation is heterozygous (Additional file 6A) or homozygous (Additional file 6B) [33]. For a given unknown mismatch site existing in multiple tissues, a PSS was given to test its probability for either a genome variation (PSS = −1, with mismatch ratio ≥ 95% or between 40% ~ 60%) or an editing (PSS = 1, with mismatches ratios between 5% ~ 40% or between 60% ~ 95%) in each sample (Figure 3A and Additional file 12). To optimize parameters for PSS cutoff by considering both efficiency of gSNPs removal and the number of nucleotide variants remained after the removal, we permuted all possible combinations among 40% ~ 60% and 90% ~ 100%. The combination of 40% ~ 60% and ≥ 95% in current analysis is among the best parameter for our purpose (Zhu, et al., unpublished data). A final overall PSS for each mismatch site was obtained by adding up PSSs from multiple tissues and cell lines. PSSs for known SNPs were calculated with a similar strategy and their distribution was then plotted against PSS from −18 to 18. With cutoff at PES < 3, over 97.5% expressed SNPs were filtered out.

### Identification of constitutive A-to-I sites in editing box regions (STEP 4)

Mismatch sites were selected using the following criteria: (i) predicted editing sites were constitutively transcribed at least from three human tissues/cell lines; (ii) each site is no longer than 50 bp away from the nearest site and the minimum transcribed genomic region is 20 bp long; (iii) Each site has a greater than 20% mismatch rate in at least one tissue; (iv) at least 5 mismatch sites clustered in one region with at least 20% conversion rate for each type of nucleotide. Thus, We named these regions containing promiscuous edited A-to-I sites as “editing boxes”.

### Characterization of constitutive A-to-I sites in editing boxes

Previously identified editing sites were retrieved from the RNA editing database (http://darned.ucc.ie/) and different studies [8-10,27] for comparison. RefSeq Genes and annotated intron/exon boundaries were retrieved from from UCSC (http://hgdownload.cse.ucsc.edu/goldenPath/hg19/database/refFlat.txt.gz). Alu and non-Alu repetitive elements were retrieved from http://hgdownload.cse.ucsc.edu/goldenPath/hg19/database/rmsk.txt.gz. IRAlus were defined as any two or more inversely oriented Alu elements located within two kilobases in their genomic location [6,12,34].

### Analyses of neighbor preferences and RNA secondary structure

Neighbor preferences were calculated based on predicted constitutive editing sites in non-repetitive or non-Alu repetitive regions, by extending 2 bases in both upstream and downstream flanking regions. The neighbor preferences were drawn by software WebLogo [35]. The structure of adjacent two editing boxes at chr2 was predicted by RNAfold from ViennaRNA Package 2.0.7 [36].

### Cell culture, plasmid construction and transfection, knockdown of ADAR1, and Western blots

HeLa cells were cultured using standard protocol provided by ATCC. Human embryonic stem cells (H9 line) were maintained as described before [37]. Sequences of editing box region (Additional file 13) were cloned into the pEGFP series vectors [12] and each plasmid was transfected into HeLa cells for 24 hours prior to harvest total RNAs for editing analysis. Sense and antisense oligonucleotides were designed based on a human ADAR1 targeting sequence (5′- GTTGACTAAGTCACATGTAAA-3′) [38] and a control scramble sequence (5′-GATGGCATTACGGCATGTTCA-3′) [39] and cloned into pLVTHM vector. Lentivirus particles were produced in HEK-293FT cells with the co-transfection of packaging vectors psPAX2 and pMD2.G. For infection, HeLa cells were incubated with concentrated viral particles at 37°C overnight and the medium was changed to fresh the next day. Infected HeLa cells were collected 72 hours later for Western blots with goat anti-ADAR1 (Santa Cruz Biotechnology).

### Total RNA isolation, RT-PCR, and Sanger sequencing validation

Total RNAs from HeLa, ADAR1 knockdown HeLa cells, transfected HeLa cells, and H9 cells were extracted with Trizol Reagent (Invitrogen) according to the manufacturer’s protocol. After treatment with DNase I (Ambion, DNA-free™ kit), the cDNA was transcribed with SuperScript II (Invitrogen) with oligo (dT) or random hexamer. Genomic DNAs were purified from both cell lines by TIANamp Genomic DNA kit (Tiangen Biotech). PCR products from cDNAs and gDNAs were amplified with primers (Additional file 13), and predicted A-to-I editing sites were validated in available cell lines with the conventional Sanger sequencing. Editing ratios of validated A-to-I sites by Sanger sequencing were calculated by “ImageJ” (http://rsb.info.nih.gov/ij/index.html). Briefly, the areas of edited and unedited signals, indicated as the signal intensities at each site, were carefully selected and measured by “ImageJ”. The editing ratio was then calculated by dividing edited intensity with total intensity at the same site. Correlation of editing ratios calculated from Sanger sequencing and RNA-seq were determined by scatter plot.

### Stranded RNA-seq analysis

Strand-specific RNA-seq libraries were prepared with prereleased Directional mRNA-seq Library Kits (Illumina) with minor modifications. Briefly, after enriched by oligo-dT selection, poly(A) + RNAs were fragmented, and treated with phosphatase and polynucleotide kinase to repair the ends. RNA adapters were sequentially ligated to the 3′ and 5′ ends of RNA fragments and reverse transcribed using a primer complementary to the 3′ linker. cDNA library was then amplified and sequenced on HiSeq2000 with 1x100 bp reads. The sequence file can be accessed from the NCBI Sequence Read Archive by GEO Accession Number GSE44450.

## Conclusions

We present an integrative approach to quantitatively profile constitutive A-to-I RNA editing from multiple human transcriptomes in the absence of the relevant genomic information. The application of our approach has allowed us to identify a large number of clustered constitutive A-to-I sites, including 695 novel ones. We further demonstrated that non-repetitive editing boxes could be promiscuously edited by ADAR1, independent of their adjacent IRAlus. Strikingly, clear differences of editing levels in the same editing box sites but from different tissues/cell-lines were also observed, strongly indicating a spatial and dynamic regulation of A-to-I RNA editing across human tissues. Our work thus offers new insights into the catalytic pattern and complex regulation of A-to-I editing by ADAR1.

## Abbreviations

EB: Editing box; ESC: Embryonic stem cell; FPKM: Fragments per kilobase per million; gDNA: Genomic DNA; gSNP: Genomic SNP; HPB: Hits per billion-mapped-bases; RPKM: Reads per kilobase per million; PSS: Potential SNP score; SNP: Single nucleotide polymorphisms.

## Competing interests

The authors declare that they have no competing interests.

## Authors’ contributions

LY and LLC conceived the study, analyzed data and wrote the manuscript from the inputs from all authors. SSZ and LY carried out the computational analyses, XJF and CT carried our all experiments. All authors have read and approved the manuscript for publication.

## Supplementary Material

Additional file 1 **Distribution of RNA-DNA mismatch ratios along the reads.** Reads from 18 of human tissues/cell lines were uniquely mapped to human reference and all types of RNA-DNA mismatches were examined at each position of 75-bp reads. Each sample was shown with different color. Click here for file

Additional file 2 Numbers of total reads used for alignment, mapped reads after the first- and second-round alignment for all transcriptomes from 18 tissues/cell lines. Click here for file

Additional file 3 **The evaluation of the two-round mapping with other aligners, SOAP **[8]**(A) and BWA **[9]**(B).** The two-round mapping approach achieved a significant increase of mapped mismatches (bottom panel) and subtle changes of mapped reads (top panel). The 1st-round mapping, light grey bars; the 2nd-round mapping, dark grey bars. Click here for file

Additional file 4 **A formula to show that normalized expression level (hits per-billion-mapped bases, HPB) of a give site is equivalent to the value of RPKM/FPKM at one nucleotide base resolution.** Click here for file

Additional file 5 **Constitutive A-to-I editing sites in editing boxes (see spreadsheet).** 2,245 constitutive A-to-I editing sites in editing boxes were listed with their genomic location, expression levels in each tissue/cell line (> 5 HPB), unique hits, editing ratios and final PSSs. Each site was also characterized with information of the overlapped gene, strand information, overlapping with RepeatMask sequences, genomic location with IRAlus and comparison with other reported editing datasets ([8-10,27] and DARNED database). Click here for file

Additional file 6 **Validation of unannotated genome variations filtered out with custom PSS cutoff.** Sanger sequencing of gDNAs and cDNAs from H9 cells were compared from randomly selected (A) heterozygous or (B) homozygous sites, which were highlighted with arrows and were proven to be real genome variations. The reference genome sequences from hg19 human genome are listed with the variation sites underlined. (C) Additional eight examples of unannotated SNPs predicted with PSS were shown with genomic locations, types of nucleotide conversion, and PSS. All of them were validated by Sanger sequencing. Three heterozygous sites (A) were only in genome 1000 dataset, but not in UCSC SNP135. All other 11 homozygous site (B and C) were not reported by either dataset. Click here for file

Additional file 7 **Tissue-specific expression of GluR-B in brain.** (A) The well characterized A-to-I editing sites at chr4:158,281,294 and chr4:158,257,875 in the pre-mRNA of GluR-B were only detected in brain, with editing frequencies at 69.1% and 91.7%, respectively, as predicted with our computational flow. (B) The expression of GluR-B in all examined samples was retrieved from UCSC genome browser and the relative expression was listed with a normalized FPKM value for each sample. Note that GluR-B is highly expressed in human tissue but few if any in other samples. Click here for file

Additional file 8 **Expression of an intergenic region with two predicted editing boxes in all 18 samples.** The expression of the intergenic region from chr2 along with its adjacent genes in all examined RNA-seq samples. The gene models, reported and predicted poly(A) sites, transcription level, and ENCODE epigenetic modifications of ChIP-seq data (H3K4Me3, H3K4Me1, H3K27Ac) were retrieved from UCSC genome browser. A new gene model of EIF2AK2 with extended 3′ UTR (red line) was drawn beneath the UCSC genome browser snapshot. Three editing boxes (two non-repetitive boxes in purple and one Alu box in pink) were indicated in the extended 3′UTR region of EIF2AK2. Note that editing boxes in this unannotated region were highly expressed in all examined samples. Click here for file

Additional file 9 **Validation of predicted A-to-I editing sites in other editing boxes.** Predicted A-to-I editing sites were highlighted in red (novel sites) or black (reported ones). Predicted editing ratios were shown above each site in the cDNA sequencing chromatograms. Validation of some A-to-I editing sites from editing boxes at (A) chr2: 37,327,644-37,327,685; (B) chr12: 69,237,506-69,237,558; (C) chr14: 23,441,376-23,441,503. Editing ratios in chr12: 69,237,529 (B) were underestimated in our analysis compared with conventional Sanger sequencing, probably due to more mismatches in short fragments failed to map to reference genome. Note that predicted sites with low editing ratio were difficult to be validated due to the limited sensitivity of the Sanger method. (D) Scatter plot of editing ratios for 31 A-to-I editing events (Figure 5B and Additional file 9A-9C) identified by RNA-seq and Sanger sequencing method. Data points corresponding to false positive or false negative predictions were shown with red dots. R, R squares and P value for the linear regression (black line) indicated the relatively good correlation between these two methods. Click here for file

Additional file 10 **Validations of A-to-I sites in editing boxes with knockdown of adar1 in HeLa cells.** Editing sites in regions chr2:37,327,656-37,327,685 (A) and chr12: 69,237,509-69,237,534 (B). Click here for file

Additional file 11 Editing ratios in three editing boxes in chromosome 2 across human tissues/cell lines. Click here for file

Additional file 12 The distribution of mismatch ratios of known genomic SNPs and predicted mismatches in all 18 samples. Click here for file

Additional file 13 **Primer sets for PCR/RT-PCR, editing box cloning and Sanger sequencing validation.** Same primer sets were used for genomic DNA and cDNA amplification unless addressed separately (−g for genomic DNA or -c for cDNA). Forward primers were chosen for Sanger sequencing. Primers for editing box cloning at ch2:37327479–37328193 region were also listed. Click here for file
