# Supplementary figures and images for: Prediction of constitutive A-to-I editing sites from human transcriptomes in the absence of genomic sequences
Source: BMC Genomics. 2013 Mar 27;14:206. doi: 10.1186/1471-2164-14-206 (PMC3637798; doi:10.1186/1471-2164-14-206)

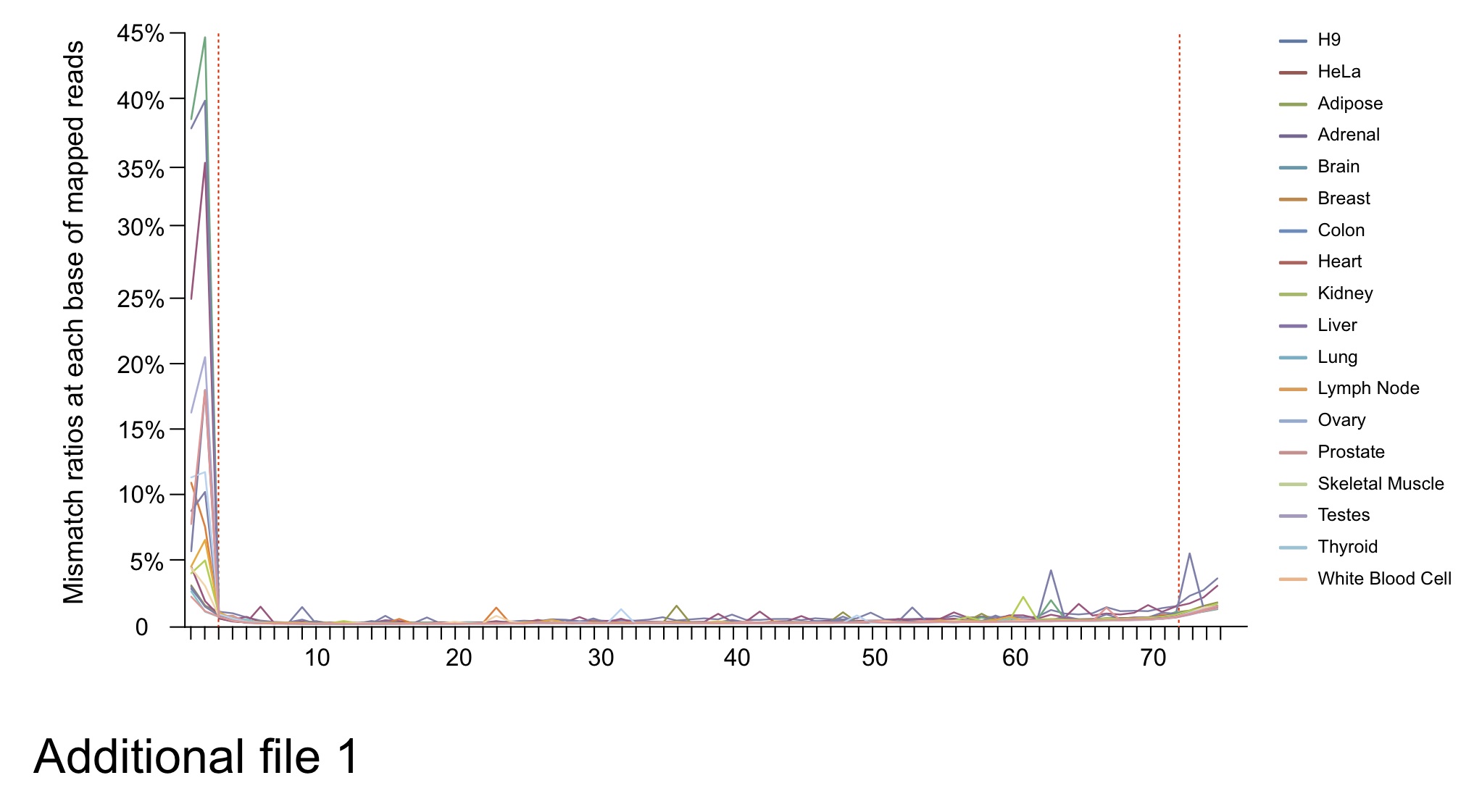

Supplement: Additional file 1 — Distribution of RNA-DNA mismatch ratios along the reads. Reads from 18 of human tissues/cell lines were uniquely mapped to human reference and all types of RNA-DNA mismatches were examined at each position of 75-bp reads. Each sample was shown with different color. [file 1471-2164-14-206-S1.jpeg]

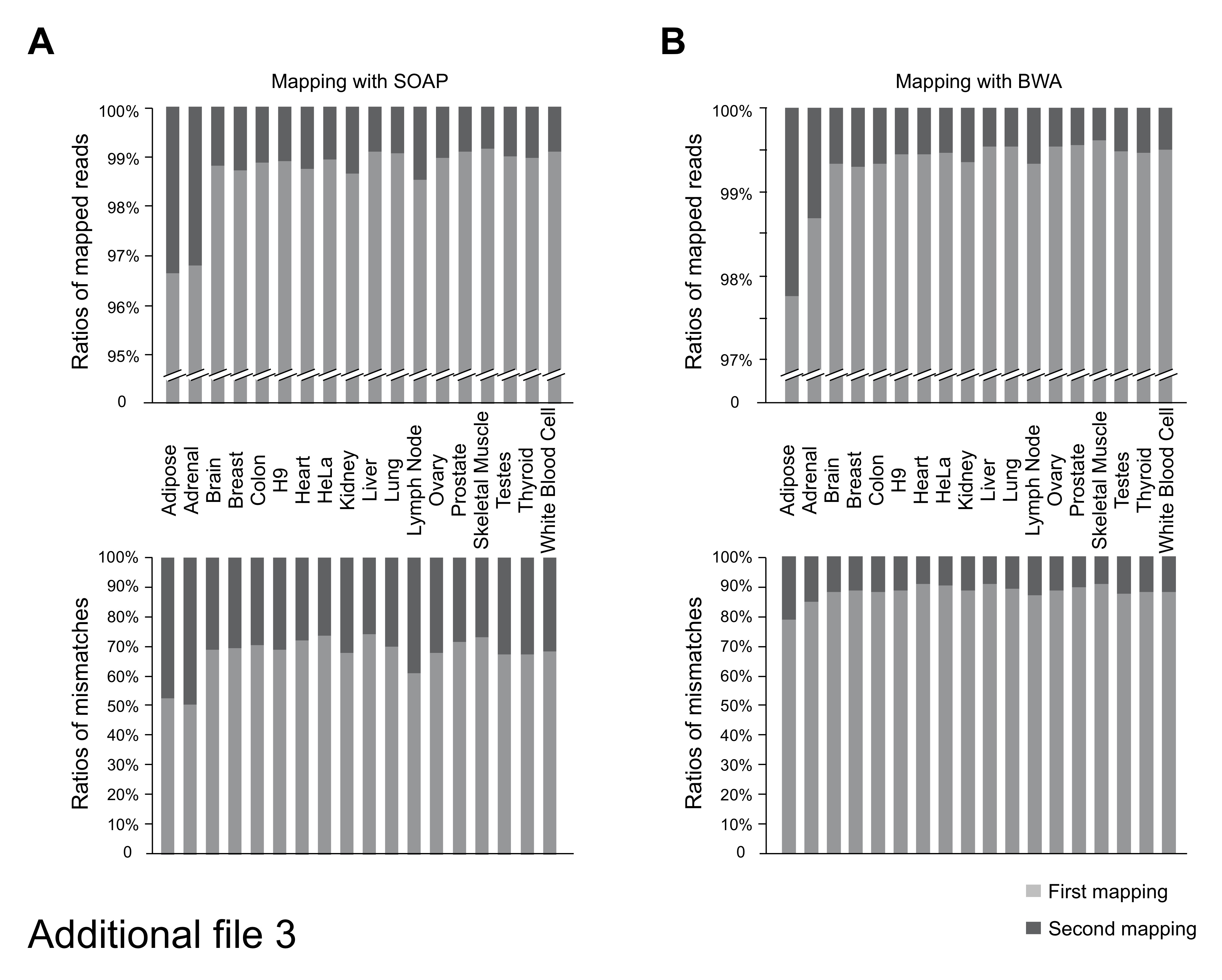

Supplement: Additional file 3 — The evaluation of the two-round mapping with other aligners, SOAP [8](A) and BWA [9](B). The two-round mapping approach achieved a significant increase of mapped mismatches (bottom panel) and subtle changes of mapped reads (top panel). The 1st-round mapping, light grey bars; the 2nd-round mapping, dark grey bars. [file 1471-2164-14-206-S3.jpeg]

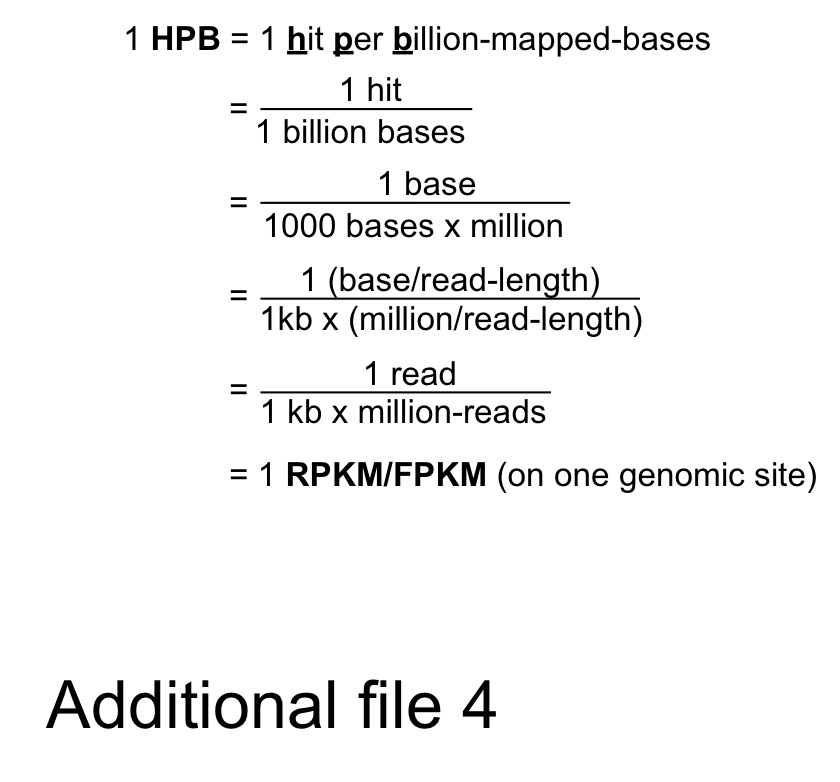

Supplement: Additional file 4 — A formula to show that normalized expression level (hits per-billion-mapped bases, HPB) of a give site is equivalent to the value of RPKM/FPKM at one nucleotide base resolution. [file 1471-2164-14-206-S4.jpeg]

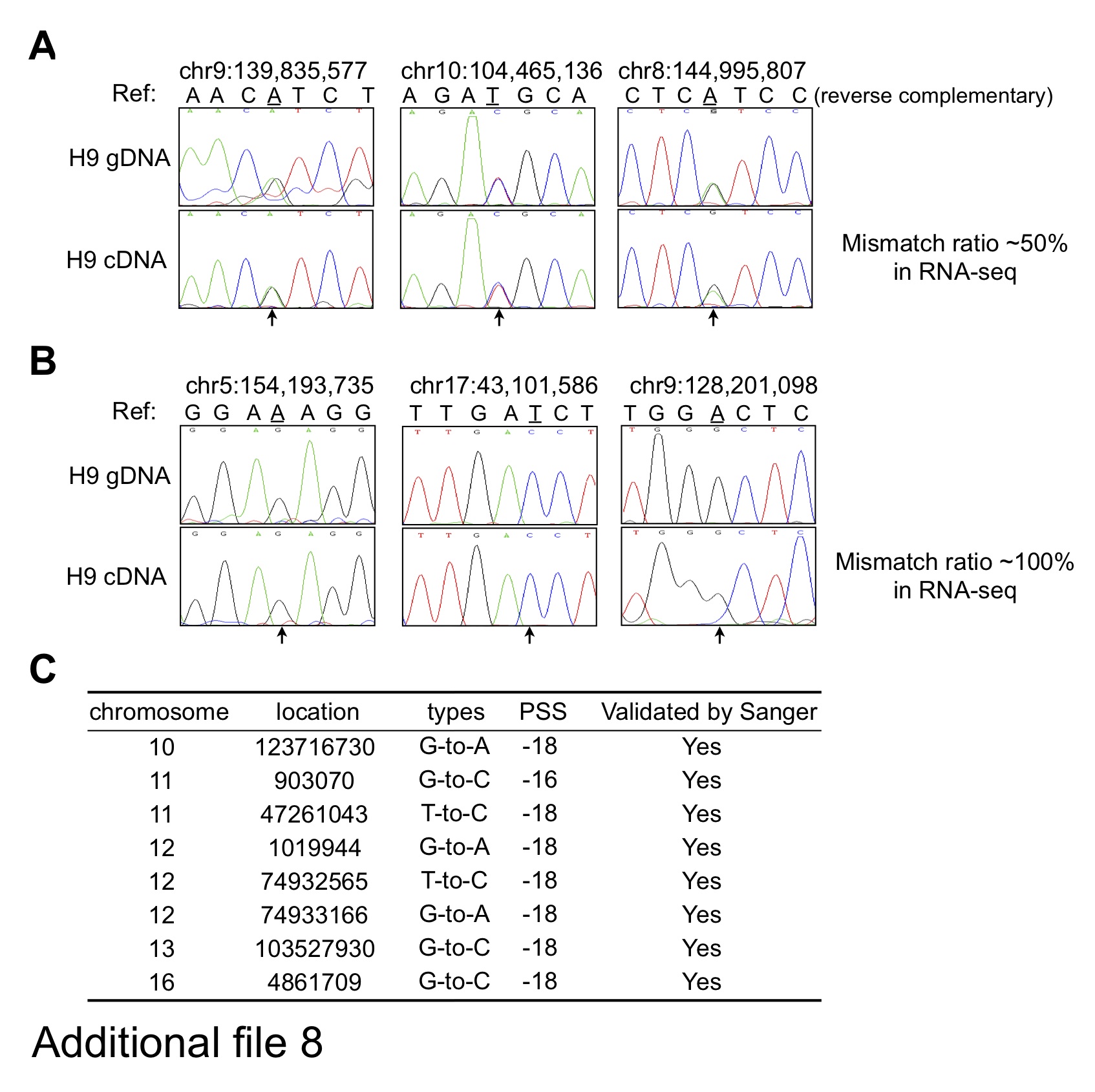

Supplement: Additional file 6 — Validation of unannotated genome variations filtered out with custom PSS cutoff. Sanger sequencing of gDNAs and cDNAs from H9 cells were compared from randomly selected (A) heterozygous or (B) homozygous sites, which were highlighted with arrows and were proven to be real genome variations. The reference genome sequences from hg19 human genome are listed with the variation sites underlined. (C) Additional eight examples of unannotated SNPs predicted with PSS were shown with genomic locations, types of nucleotide conversion, and PSS. All of them were validated by Sanger sequencing. Three heterozygous sites (A) were only in genome 1000 dataset, but not in UCSC SNP135. All other 11 homozygous site (B and C) were not reported by either dataset. [file 1471-2164-14-206-S6.jpeg]

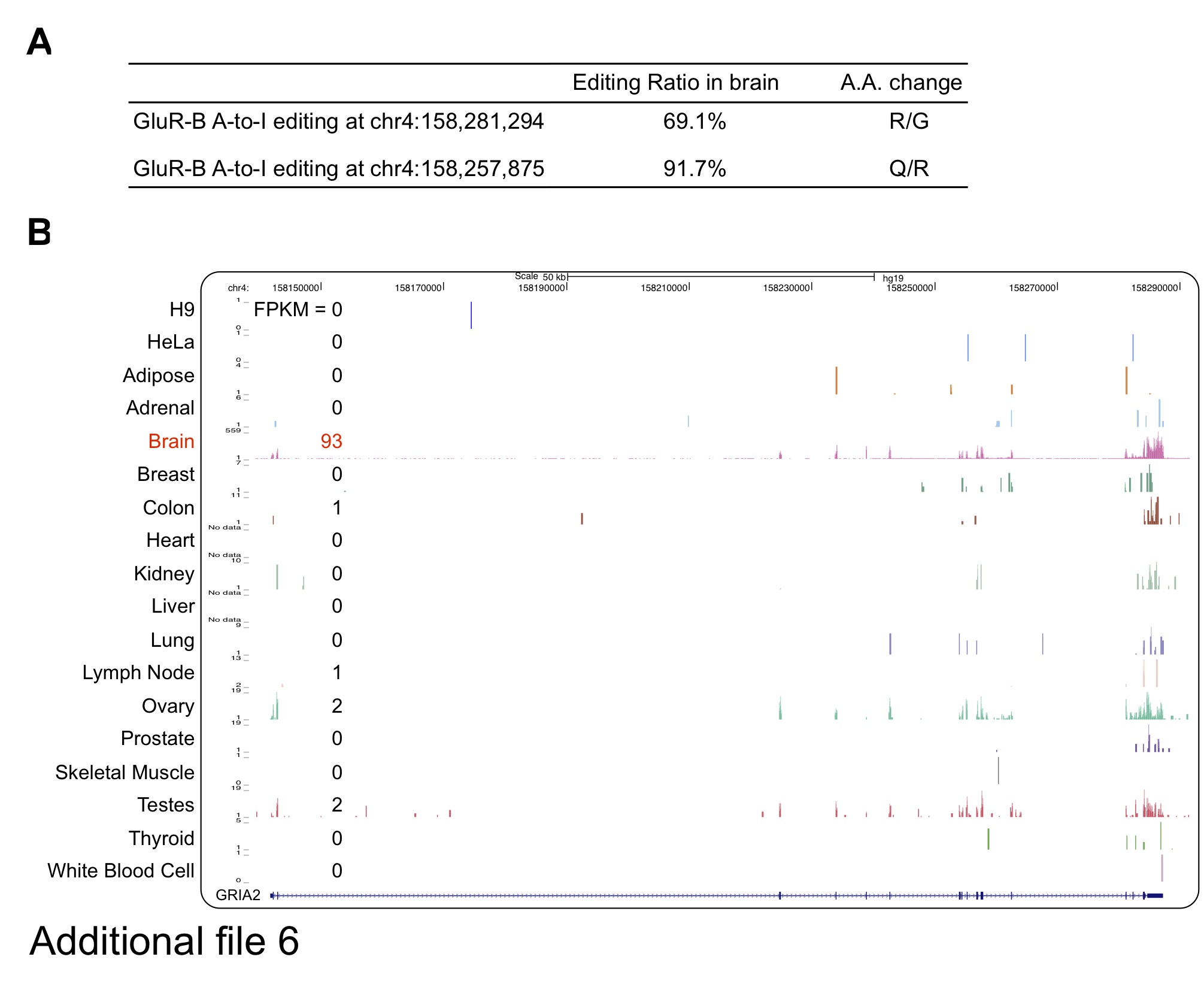

Supplement: Additional file 7 — Tissue-specific expression of GluR-B in brain. (A) The well characterized A-to-I editing sites at chr4:158,281,294 and chr4:158,257,875 in the pre-mRNA of GluR-B were only detected in brain, with editing frequencies at 69.1% and 91.7%, respectively, as predicted with our computational flow. (B) The expression of GluR-B in all examined samples was retrieved from UCSC genome browser and the relative expression was listed with a normalized FPKM value for each sample. Note that GluR-B is highly expressed in human tissue but few if any in other samples. [file 1471-2164-14-206-S7.jpeg]

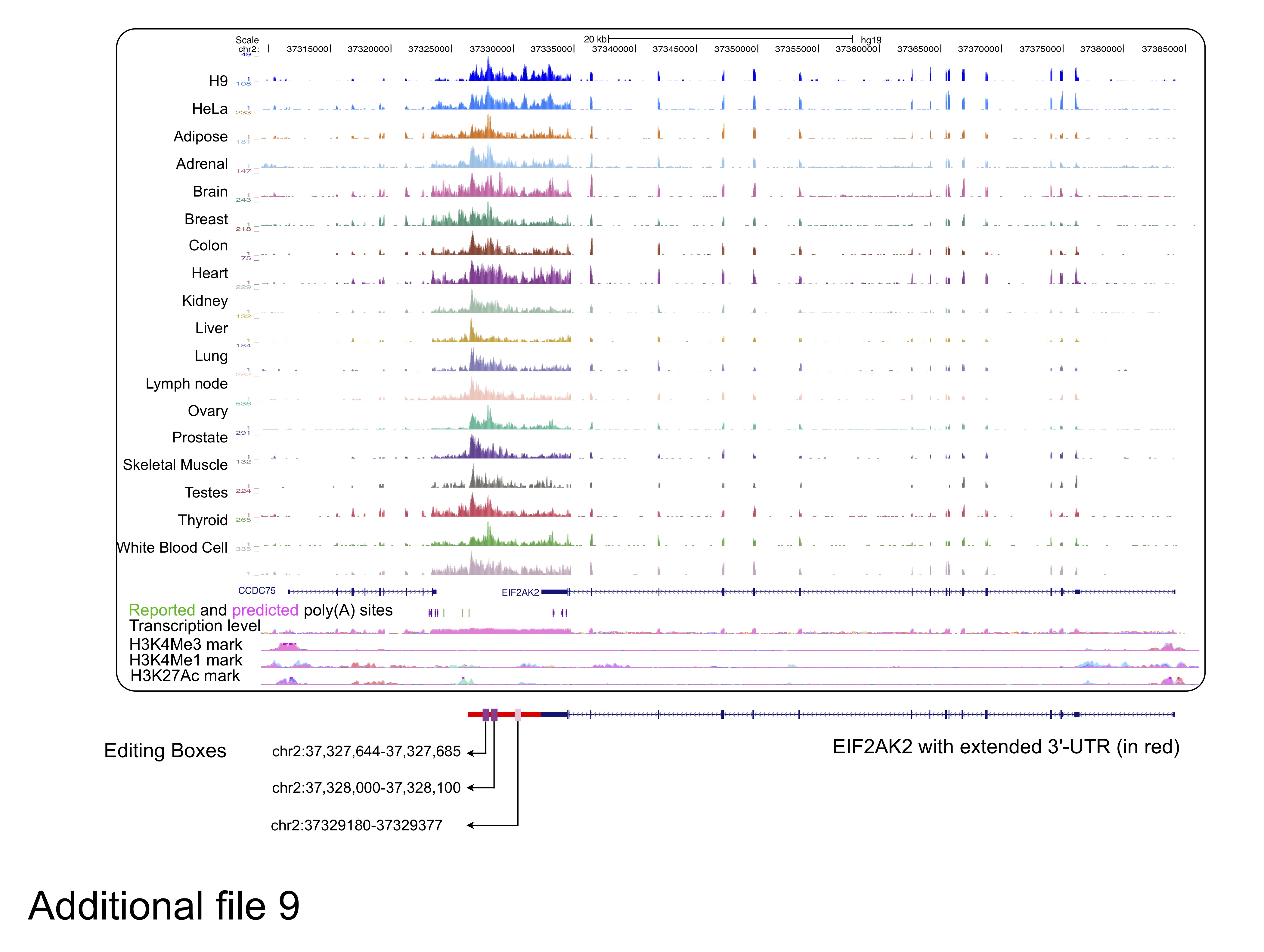

Supplement: Additional file 8 — Expression of an intergenic region with two predicted editing boxes in all 18 samples. The expression of the intergenic region from chr2 along with its adjacent genes in all examined RNA-seq samples. The gene models, reported and predicted poly(A) sites, transcription level, and ENCODE epigenetic modifications of ChIP-seq data (H3K4Me3, H3K4Me1, H3K27Ac) were retrieved from UCSC genome browser. A new gene model of EIF2AK2 with extended 3′ UTR (red line) was drawn beneath the UCSC genome browser snapshot. Three editing boxes (two non-repetitive boxes in purple and one Alu box in pink) were indicated in the extended 3′UTR region of EIF2AK2. Note that editing boxes in this unannotated region were highly expressed in all examined samples. [file 1471-2164-14-206-S8.jpeg]

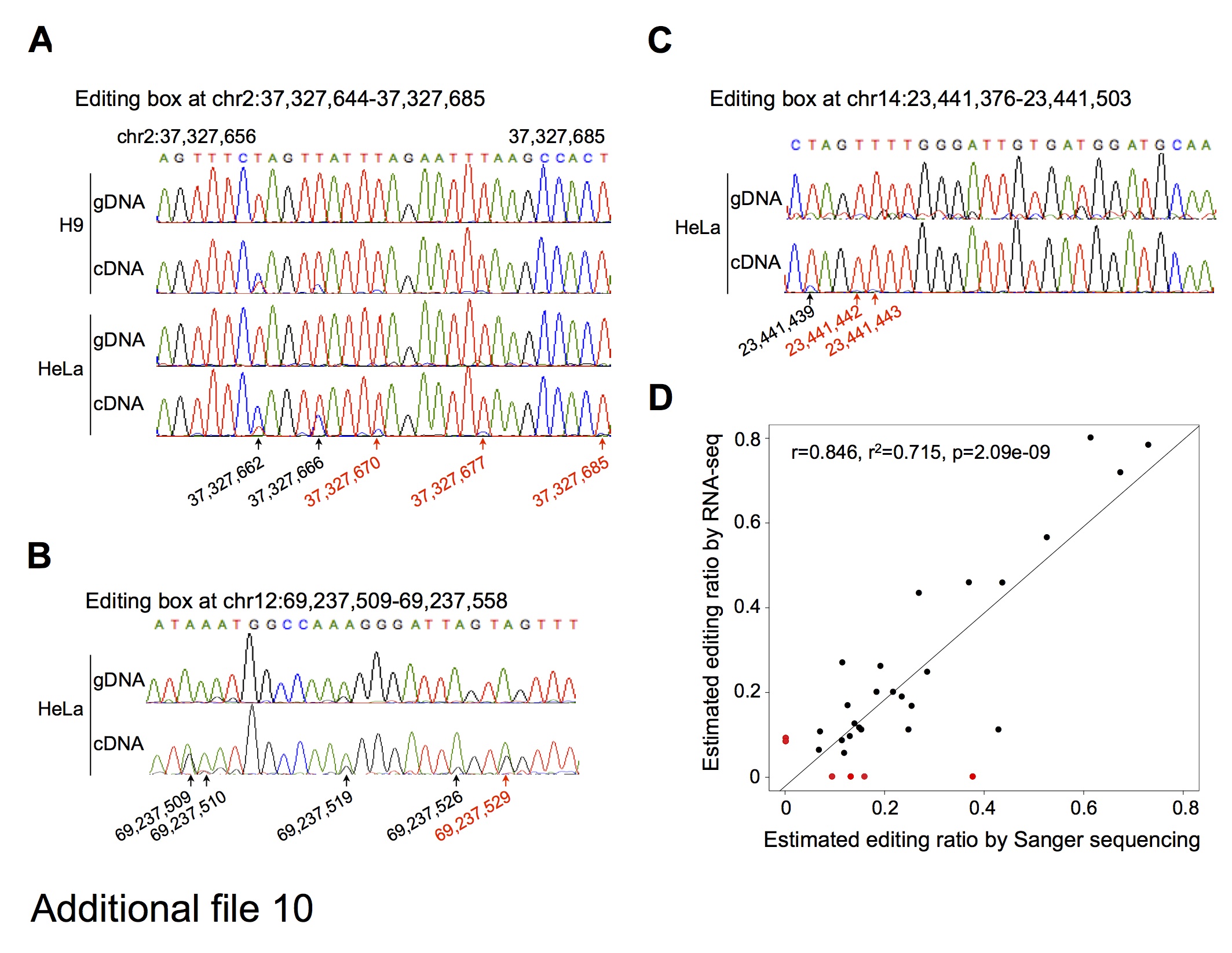

Supplement: Additional file 9 — Validation of predicted A-to-I editing sites in other editing boxes. Predicted A-to-I editing sites were highlighted in red (novel sites) or black (reported ones). Predicted editing ratios were shown above each site in the cDNA sequencing chromatograms. Validation of some A-to-I editing sites from editing boxes at (A) chr2: 37,327,644-37,327,685; (B) chr12: 69,237,506-69,237,558; (C) chr14: 23,441,376-23,441,503. Editing ratios in chr12: 69,237,529 (B) were underestimated in our analysis compared with conventional Sanger sequencing, probably due to more mismatches in short fragments failed to map to reference genome. Note that predicted sites with low editing ratio were difficult to be validated due to the limited sensitivity of the Sanger method. (D) Scatter plot of editing ratios for 31 A-to-I editing events (Figure 5B and Additional file 9A-9C) identified by RNA-seq and Sanger sequencing method. Data points corresponding to false positive or false negative predictions were shown with red dots. R, R squares and P value for the linear regression (black line) indicated the relatively good correlation between these two methods. [file 1471-2164-14-206-S9.jpeg]

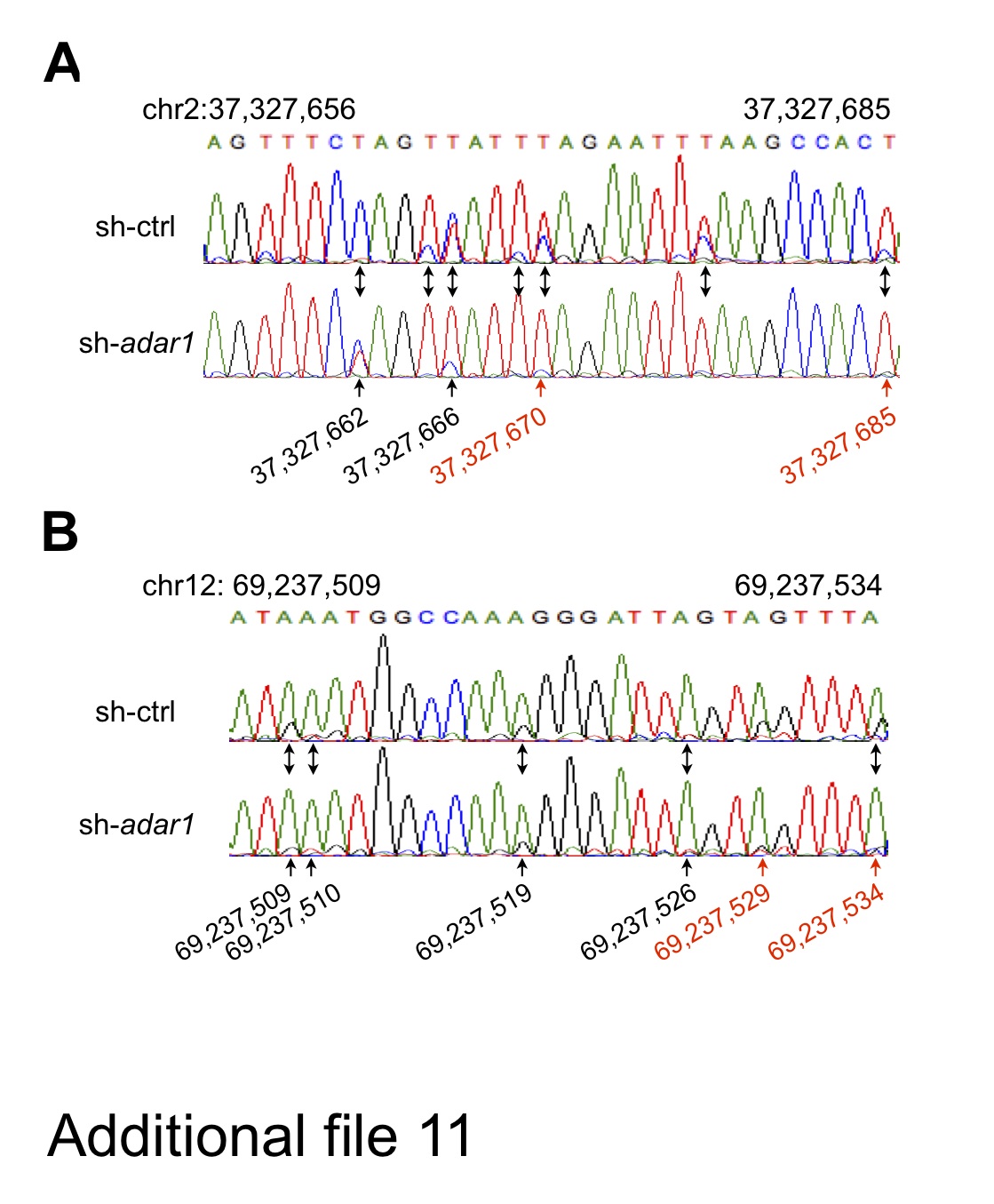

Supplement: Additional file 10 — Validations of A-to-I sites in editing boxes with knockdown of adar1 in HeLa cells. Editing sites in regions chr2:37,327,656-37,327,685 (A) and chr12: 69,237,509-69,237,534 (B). [file 1471-2164-14-206-S10.jpeg]

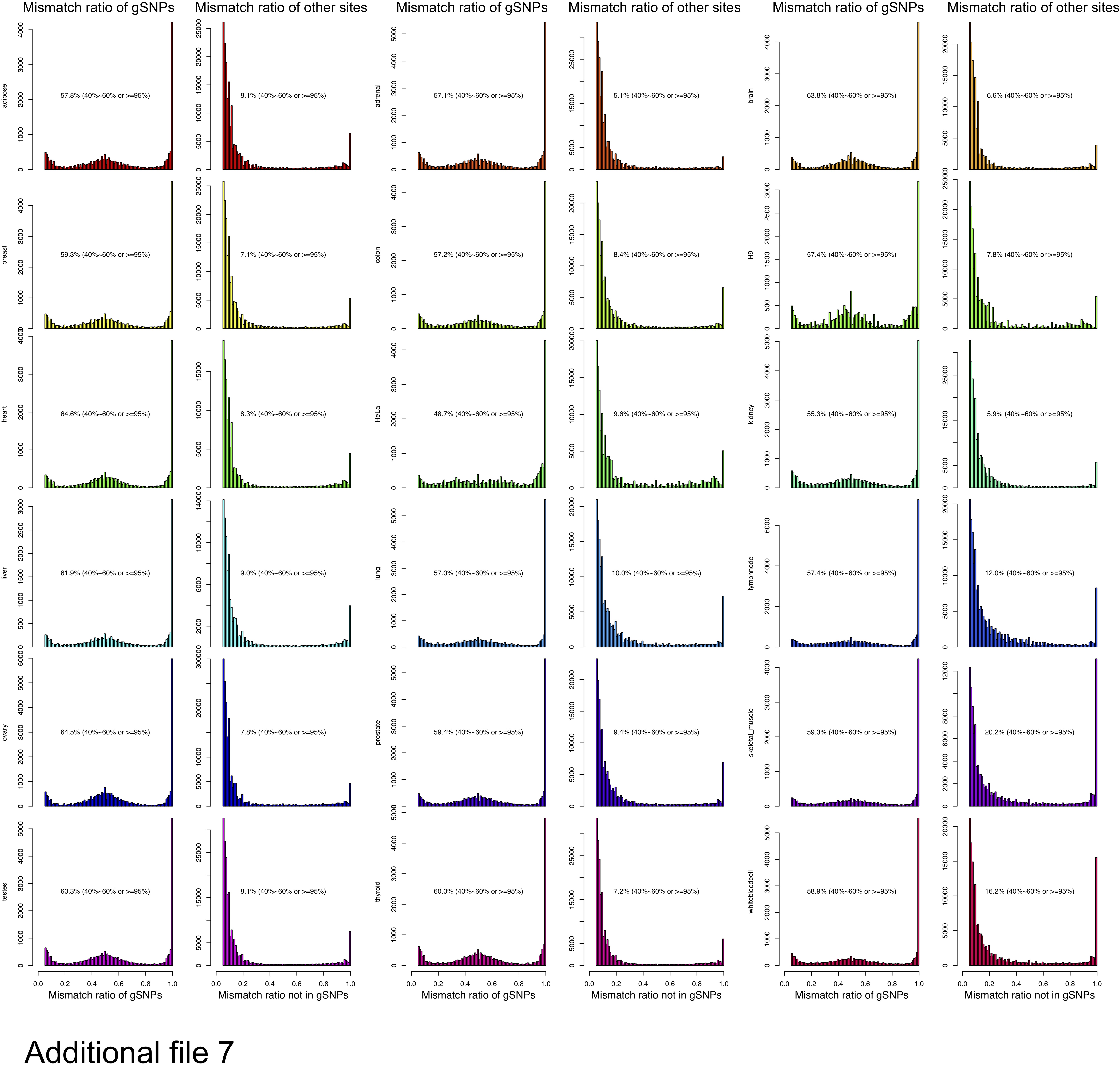

Supplement: Additional file 12 — The distribution of mismatch ratios of known genomic SNPs and predicted mismatches in all 18 samples. [file 1471-2164-14-206-S12.jpeg]

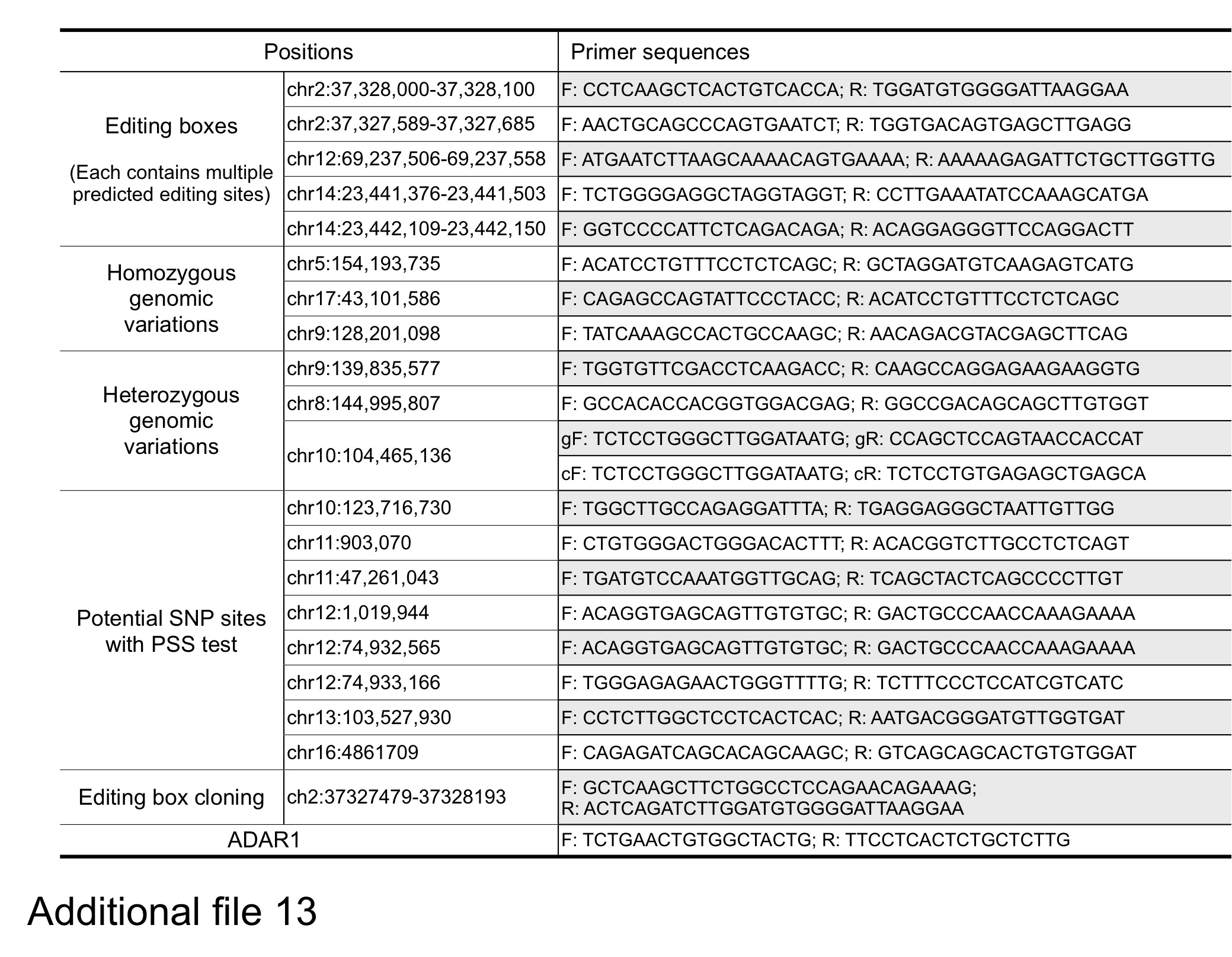

Supplement: Additional file 13 — Primer sets for PCR/RT-PCR, editing box cloning and Sanger sequencing validation. Same primer sets were used for genomic DNA and cDNA amplification unless addressed separately (−g for genomic DNA or -c for cDNA). Forward primers were chosen for Sanger sequencing. Primers for editing box cloning at ch2:37327479–37328193 region were also listed. [file 1471-2164-14-206-S13.jpeg]
